# Phage-antibiotic combination: a possible approach to combatting multidrug-resistant *Klebsiella pneumoniae*

**DOI:** 10.1186/s13568-025-02005-1

**Published:** 2026-03-25

**Authors:** Nourhan I. Abdelhakim, Walaa A. Alshareef, Sarra E. Saleh, Mahmoud A. Yassein

**Affiliations:** 1 Department of Microbiology and Immunology, Faculty of Pharmacy, October 6 University, Sixth of October City, 12585 Giza Egypt; 2 Department of Microbiology and Immunology, Faculty of Pharmacy, Ain Shams University, Organization of African Unity Street, Abbasia, 11566 Cairo Egypt

**Keywords:** *Klebsiella pneumoniae*, Multi-drug resistant, Phage-antibiotic combination

## Abstract

Multidrug-resistant *Klebsiella pneumoniae* is a major global health concern due to its role in severe hospital-acquired infections and resistance to multiple antibiotics. As conventional antibiotics lose effectiveness, bacteriophage (phage) therapy offers a promising alternative. This study assessed phage therapy alone and combined with antibiotics against multidrug-resistant *K. pneumoniae* using the time-kill assay. A total of 48 clinical isolates were collected and identified as *K. pneumoniae.* According to their antibiotic susceptibility, 91.7% (n = 44) of the tested isolates were multidrug-resistant. Sewage samples were processed to isolate a phage-coded Kp25, which showed lytic activity against 17 isolates and remained stable within a temperature range of 40–90 °C and a pH range of 3–11. The taxonomic classification of the phage was made clear by the findings of the genomic analysis, which is *Viruses; Duplodnaviria; Heunggongvirae; Uroviricota; Caudoviricetes; Drexlerviridae; Webervirus; Webervirus domnhall*. Phage combination with sub-MIC of levofloxacin and meropenem showed potential antibacterial activity, so that complete in vitro bacterial eradication was achieved starting from 4 h incubation until the end of the incubation period. However, phage combination with sub-MIC of gentamicin showed weak antibacterial activity. These findings highlight the phage combination with levofloxacin and meropenem as an effective strategy against multidrug-resistant *K. pneumoniae*.

**Supplementary Information:**

The online version contains supplementary material available at 10.1186/s13568-025-02005-1.

## Introduction

Microorganisms continue to demonstrate their resiliency in ways that are unanticipated in the continuous competition between diseases and scientific research. The rise in incidence of multi-drug resistant (MDR) bacterial infections exhibits a formidable challenge to global public health and clinical medicine (Kumar et al. 2024). In a recent warning, the World Health Organization (WHO) expressed concern that the development of antibiotic resistance could become one of the most prevalent causes for death by 2050 if left unchecked (Turkey and Nazeam 2025). Beyond diminishing antibiotic efficacy, excessive antibiotic use disrupts the human microbiome, particularly gut flora, further exacerbating health complications (Ebrahim et al. 2022).

Despite advances in bioinformatics and targeted drug development, MDR infections remain a critical unresolved issue worldwide (Mallari et al. 2025). Among these, *Klebsiella pneumoniae,* a non-motile, Gram-negative bacterium that belongs to the family *Enterobacteriaceae,* stands out as a formidable opportunistic pathogen. Capable of metabolizing diverse substrates, *K. pneumoniae* is associated with nosocomial infections, including pneumonia, urinary tract infections, and wound infections. Hypervirulent strains can cause serious diseases such as liver abscesses, necrotizing soft tissue infections, and bacteremia (Aslam et al. 2022). The WHO has classified MDR *K. pneumoniae* as a pathogen of high priority with epidemic potential in healthcare settings due to its association with elevated morbidity and mortality rates (Christaki et al. 2020). *K. pneumoniae* has evolved many mechanisms to resist the antimicrobial agents. These resistance mechanisms involve the production of degrading enzymes, alteration of active site conformation, multiplication of target sites, and inhibition of intracellular antibiotic accumulation by reducing influx or enhancing efflux via efflux pumps (Abdel-Halim et al. 2022).

There is a story of survival, adaptation, and the necessity of more intelligent treatments associated with each and every resistant strain. Natural environments, particularly wastewater, serve as rich reservoirs for novel antimicrobial agents. As a convincing alternative to antibiotics, bacteriophages (phages), viruses that are capable of infecting and lysing bacterial cells, have recently come back into the spotlight. (Ragab et al. 2024). Phage therapy predates antibiotics, with historical use in treating bacterial infections via oral, topical, or systemic administration (Li et al. 2020). Some phages exploit bacterial polysaccharides as receptors, employing specialized tail structures and depolymerases to penetrate biofilms and infect resistant strains (Islam et al. 2024; Volozhantsev et al. 2022).

Despite these advances, the influence of phage therapy on MDR *K. pneumoniae*’s virulence and resistance profiles remains underexplored. Limited research exists on phage-mediated suppression of bacterial proliferation, leaving a critical gap in antimicrobial development. This study addresses this gap by evaluating phage therapy’s standalone and in combination with sub-inhibitory concentrations of antibiotics. Our findings aim to elucidate phage-antibiotic combinations as a viable, cost-effective strategy for managing MDR *K. pneumoniae* infections, paving the way for future clinical applications.

## Materials and methods

### Isolates collection and identification

*Klebsiella* spp. isolates were collected between March 2023 and July 2023 at the Diagnostic Microbiology Laboratories of October 6 University Hospital and Al-Qasr Al-Ainy Hospital, Cairo, Egypt. They were isolated from clinical specimens that include urine, blood, and sputum.

The bacterial isolates were initially cultivated on nutrient agar (Cat. No. M001, HiMedia, Mumbai, India) and then streaked onto MacConkey agar plates (Cat. No. M081, HiMedia, Mumbai, India). They were incubated at 37°C for 24 h to allow colony formation, which was subsequently evaluated based on distinctive features, including lactose fermentation and mucoid texture commonly associated with *Klebsiella* species (Patel et al. 2017).

Preliminary identification involved microscopic examination through Gram staining, along with a series of biochemical assays, such as Indole, Methyl Red (MR), Voges-Proskauer (VP), and Citrate Utilization Test (IMViC). To confirm species-level identification, isolates were subjected to analysis using the Vitek 2 Compact automated system (bioMérieux, France), following the provided standard operating procedure. Following the identification of the clinical isolates, they were stored in Luria–Bertani (LB) broth (Cat. No. M1245, HiMedia, Mumbai, India), where they were kept at a temperature of – 80°C for later uses (Abbas et al. 2020).

*K. pneumoniae* clinical isolate K37 was formally deposited in the Culture Collection of Ain Shams University (CCASU-2025-80) that is registered under the World Data Centre for Microorganisms (WDCM) (10.12210/ccinfo.1186) as a representative sample and host for the phage Kp25.

### Molecular characterization of bacterial isolate

#### DNA extraction

Genomic DNA of the K37 isolate was extracted utilizing the QIAamp DNA Mini Kit (Qiagen, Cat. No. 51304, Hilden, Germany) in accordance with the manufacturer's instructions.

#### PCR amplification of 16S rRNA

The 16S rRNA gene was amplified with primers F27 (5′-AGAGTTTGATCMTGGCTCAG-3′) and R1492 (5′-TACGGYTACCTTGTTACGACTT-3′) (Metabion, Germany). A 25 μl reaction mixture including 12.5 μl of Emerald Amp GT PCR Master Mix (Takara, Code No. RR310A, Japan), 1 μl of each primer, 5 μl template DNA, and 5.5 μl PCR-grade water was used for PCR amplification in a Biometra T3 thermal cycler (Biometra, Germany). Amplification products were analyzed by 1% agarose gel electrophoresis (ABgene, UK) prepared in TBE buffer (Fluka, Switzerland), stained with ethidium bromide (Sigma-Aldrich, USA), and visualized using a gel documentation system (Alpha Innotech, USA).

#### PCR product purification and sequencing

Amplicons were purified with the QIAquick PCR Product Extraction Kit (Qiagen, USA, Cat. No. 28104). Sequencing was performed in both forward and reverse orientations using the BigDye Terminator v3.1 Cycle Sequencing Kit (Applied Biosystems, USA; Cat. No. 4336817). Reactions were purified utilizing the Centri-Sep Spin Column Kit (Princeton Separations, USA) and subsequently analyzed on an Applied Biosystems 3130 Genetic Analyzer (Hitachi, Japan).

The acquired 16S rRNA sequence was analyzed against reference sequences in the NCBI GenBank database with the BLASTn program (Altschul et al. 1990) to verify the identity of the isolate.

#### Antimicrobial susceptibility testing

The Kirby-Bauer disk diffusion technique was utilized on Mueller–Hinton Agar (MHA) (Cat. No. M173, HiMedia, Mumbai, India) in order to carry out the antibiotic susceptibility testing of the clinical *K. pneumoniae* following the guidelines established by Clinical and Laboratory Standards Institute (CLSI 2021). The turbidity of the bacterial suspension was carefully adjusted to match approximately 1 × 10^8^ CFU/mL before application and lawn-cultured onto MHA plates (Shilpa et al. 2016). Antibiotic discs were aseptically applied, including Imipenem (10 μg), Meropenem (10 μg), Gentamicin (10 μg), Amikacin (30 μg), Streptomycin (25 μg), Cotrimoxazole (25 μg), Aztreonam (30 μg), Tobramycin (10 μg), Ciprofloxacin (5 μg), Levofloxacin (5 μg), Ticarcillin/Clavulanic acid (85 μg), Cefotaxime (30 μg), Cefuroxime (30 μg), Ceftazidime (30 μg), Cefepime (30 μg), and Ceftriaxone (30 μg) (Bioanalyse, Ankara, Turkey). Zones of inhibition were measured following the incubation period of 24 h at 37 °C (Qadri et al. 2021).

#### Determination of minimum inhibitory concentration

The determination of MIC was carried out via the broth microdilution technique in 96-well plates. A stock solution of each antibiotic was initially prepared to achieve a final concentration of 2000 μg/mL. Different concentrations of the antibiotics were prepared through serial dilution of the stock solution in Mueller–Hinton Broth (MHB) (Cat. No. M391, HiMedia, Mumbai, India). Bacterial inocula (5 × 10^5^ CFU/mL) were added, and plates were incubated at 37 °C for 24 h. MIC testing was conducted for three antibiotics: levofloxacin, meropenem, and gentamicin against three multidrug-resistant *K. pneumoniae* isolates (K22, K35, and K37)**.** MIC was defined as the lowest concentration that prevented visible bacterial growth (Rezk et al. 2022).

For both antimicrobial susceptibility testing and determination of MICs, *K. pneumoniae* ATCC 700603 was used as a reference strain.

#### Phage enrichment, isolation and purification

Twenty samples of sewage water were collected from different locations across Egypt. 4.5 mL of sewage samples were combined with 0.5 mL of an overnight *K. pneumoniae* culture and 5 mL of freshly prepared double-strength tryptone soya broth within a Falcon tube. This was done in order to enhance the phage concentration in the sewage sample. This mixture was incubated in an incubator shaker at 37 °C and 150 rpm for 24–48 h. Following enrichment, the cultures were subjected to centrifugation at 9000 ×*g* for a duration of 10 min at a temperature of 4 °C to pellet the bacterial cells. A 0.45 μm syringe filter (Cat No. SCA020025K-S, CHMLAB, Terrassa, Barcelona, Spain) was employed to filtrate the supernatant to eliminate residual bacterial debris. To confirm the presence of the lytic phage, a spotting technique was conducted by applying 10 μL of the phage lysate onto Tryptic Soy Agar (TSA) plates (Cat. No. CM0356, Techno Pharmchem, Bahadurgarh, India) previously seeded with *K. pneumoniae* and incubated for 24 h at 37 °C; a clear spot indicated lytic activity (Muangman and Gatedee 2022).

The double-layer agar (DLA) technique was performed for phage purification. Briefly, 100 μL of an overnight *K. pneumoniae* culture was mixed with 5 mL of molten top agar and 100 μL of phage lysate, then poured onto a TSA plate. The plate was subsequently incubated after solidification for 24 h at 37 °C. Discrete plaques were selected for purification.

Phage titer (plaque-forming units per mL, PFU/mL) was determined via serial dilution and the DLA method, with counts recorded for dilutions yielding 30–300 plaques per plate (Balcão et al. 2022). Aliquots of the phage lysate were stored at -80 for further microbiological assays to be conducted.

The isolated phage Kp25 was officially deposited in the Culture Collection of Ain Shams University under accession number (CCASU-2025-7-phage1) in the World Data Centre for Microorganisms (WDCM) (10.12210/ccinfo.1186). It is through this legal registration that the phage will be preserved for an extended period of time and made available to people all around the world for future reference, reproducibility, and possible applications in research or therapy.

### Characterization of isolated bacteriophages

#### Plaques morphology

To define the morphology of the formed plaques, measurements were taken to determine their diameter, clearance, and whether a halo zone was present around the plaques.

#### Determination of the host range

In the middle of plates that contained tryptone soy DLA and were inoculated with one of the multi-drug-resistant *K. pneumoniae* isolates, 10 μL of the phage sample was successfully located. After incubation for 24 h at 37 °C, the appearance of distinct zones indicated that phage activity was present against the bacteria that were tested (Chen et al. 2023).

#### Effect of the temperature on phage stability

To assess the thermal stability, the phage lysate of 5 × 10^12^ PFU/mL was incubated at varying degrees of temperature (40, 50, 70, 90, and 100°C) for one hour. After heat exposure, spot assays were performed to determine the viability of the phage by applying 10 μL of the phage lysate onto *K. pneumoniae*-seeded TSA plates. An untreated phage lysate (not exposed to heat) was performed as a positive control, whilst plates containing only bacteria were used as negative controls. The positive control exhibits a distinct lysis zone, and the negative control demonstrates an absence of lysis. The experiment was conducted in three independent replicates, and the presence of lysis zones was observed (El-Atrees et al. 2022).

#### Effect of pH on phage stability

Using either 0.1 M hydrochloric acid or sodium hydroxide, the pH of the lysate of 5 × 10^12^ PFU/mL was adjusted to values ranging from 1 to 12 to determine the stability of the phage across a variety of pH conditions. Following the incubation of the samples at 37°C for one hour. The test was performed in three independent replicates; the lytic activity of the samples was evaluated by spotting 10 μL of the phage lysate on TSA plates that had been inoculated with *K. pneumoniae.* An untreated phage lysate (not exposed to different pH) functioned as a positive control, while plates containing just bacteria only and buffer-only spots were used as negative controls. The controls served as expected, with positive controls exhibiting distinct lysis zones and negative controls displaying no lysis (Mahmoud et al. 2021).

#### Longevity test

An aliquot of the phage lysate of 5 × 10^12^ PFU/mL was stored at temperatures of 4 and − 20°C, and the lytic activity of the phage was assessed by spotting 10 μL of the phage lysate on TSA plates that had been inoculated with *K. pneumoniae* at intervals of 5, 15, 30, 45, and 60 days. The fresh phage lysate performed as a positive control, while bacteria-only plates were used as negative controls. These controls behaved as expected, with positive controls showing clear lysis zones and negative controls showing no lysis. The test was performed in three independent replicates; this was done to evaluate the storage stability (Mahmoud et al. 2021).

### Transmission electron microscopy

Phage morphology was visualized through transmission electron microscopy (TEM) using a JEM-1400 (JEOL Ltd., Tokyo, Japan) instrument running at an accelerating voltage of 80 kV. For sample preparation, the purified phage Kp25 particles were carefully placed on carbon-coated copper grids to allow adsorption. The grids were subsequently subjected to negative staining with 2% phosphotungstic acid at pH 7.0 for approximately 20 s, a step that improves contrast by characterizing the virion architecture. The sample was examined after complete drying under the electron microscope, enabling detailed observation of the phage’s structural features, including head shape and tail length (Nazir et al. 2022).

## Molecular analysis of bacteriophage

### DNA extraction

DNA was extracted from lysate using the Phage DNA Isolation Kit (Norgen, Cat. No. 46800) according to manufacturer instructions.

### Library preparation and sequencing

DNA was quantified utilizing Qubit 4 (Thermo Fischer Scientific), and the Rapid Sequencing DNA V14 barcoding kit (SQK‐RBK114.24; Oxford Nanopore Technologies, Oxford, UK) was used according to manufacturer instructions for library preparation using approximately 200 ng of the extracted DNA. MinION™ and R10.4.1 flow cells (FLO‐MIN114; Oxford Nanopore Technologies) were used for sequencing. MinKNOW software ver. 24.11.10 (Oxford Nanopore Technologies) was employed for data acquisition.

### Bioinformatics analysis

Raw sequencing data (POD5 files) were basecalled using Dorado (v7.6.8) and demultiplexed. Kraken2 was used for taxonomic classification (Wood et al. 2019) with a custom viral database, and abundance estimates were generated with Bracken (Lu et al. 2017). Reads were assembled into a draft genome using Medaka, and the assembly was annotated and compared to reference genomes using PhageScope (Wang et al. 2024).

### Multiplicity of infection optimization

The optimal multiplicity of infection (MOI) of the phage was determined by mixing 100 μL of bacterial suspension (5 × 10^5^ CFU/mL) with 100 μL of various dilutions of the phage lysate in a 96-well microtiter plate. A microplate ELISA reader (ELX800, BioTek Instruments, USA) was used to take readings of the optical density (OD600) at intervals of 0, 2, 4, 8, and 24 h. Control wells contained only the host strain. The test was performed in triplicate. The mean and standard deviation (SD) were calculated across biological replicates (Rastegar et al. 2024).

### Time-kill curve analysis

The synergistic activity between the isolated phage Kp25 and selected antibiotics (levofloxacin, gentamicin, and meropenem) was investigated through time-kill assays. Antibiotics were applied at sub-inhibitory concentrations, specifically at ¼ and ½ of their respective minimum inhibitory concentrations (MICs). Experimental conditions were established in test tubes as follows: (i) bacterial suspension alone (5 × 10^5^ CFU/mL) as a positive control, (ii) bacteria with antibiotics at sub-MIC levels, (iii) bacteria with phage at a multiplicity of infection (MOI) of 100, and (iv) bacteria with both phage (MOI 100) and antibiotics (¼ or ½ MIC). All setups were incubated at 37°C, and viable bacterial counts were assessed at 0, 2, 4, 8, and 24 h using the standard colony counting technique. After 24-h incubation at 37°C, bacterial colonies were enumerated, and results were presented as log₁₀ CFU/mL. The experiment was performed using three independent clinical isolates, with three technical replicates per isolate. The mean and standard deviation (SD) were calculated across biological replicates. Synergy was defined as a reduction of ≥ 2 log_10_ CFU/mL compared to the most active single treatment after 24 h (Rastegar et al. 2024).

### Statistical analysis

Statistical analysis was conducted utilizing IBM SPSS Statistics software, version 20.0 (IBM Corp., Armonk, NY, 2011). The Shapiro–Wilk test was utilized to evaluate the normality of data distribution. Quantitative results were presented as mean values with standard deviations (mean ± SD). Group differences were assessed via one-way ANOVA analysis of variance. To evaluate pairwise differences between groups, Tukey’s multiple comparison test was applied. Statistical significance was determined at a value of *p* < 0.05.

## Results

### Bacterial isolates

48 clinical *Klebsiella* isolates were recovered from the clinical specimens as follows: 30 from urine, 15 from sputum, and 3 from bloodstream infections. Species identification conducted via the automated Vitek 2 Compact System (bioMérieux, France) and standard microbiological techniques verified that all isolates were *Klebsiella pneumoniae*.

### Molecular characterization of bacterial isolate

The analysis of the 16S rRNA gene of isolate K37 indicated that it is *Klebsiella pneumoniae*. Sequencing produced a linear DNA fragment of 1415 bp, which was submitted to NCBI GenBank under accession number PX247847 (https://www.ncbi.nlm.nih.gov/nuccore/PX247847). BLASTn analysis against reference sequences in the NCBI database demonstrated a sequence identity exceeding 99% with *K. pneumoniae* strains, thereby validating the identity of the isolate.

### Determination of the sensitivity of the collected isolates to different types of antimicrobial agents

**Fig. 1:**
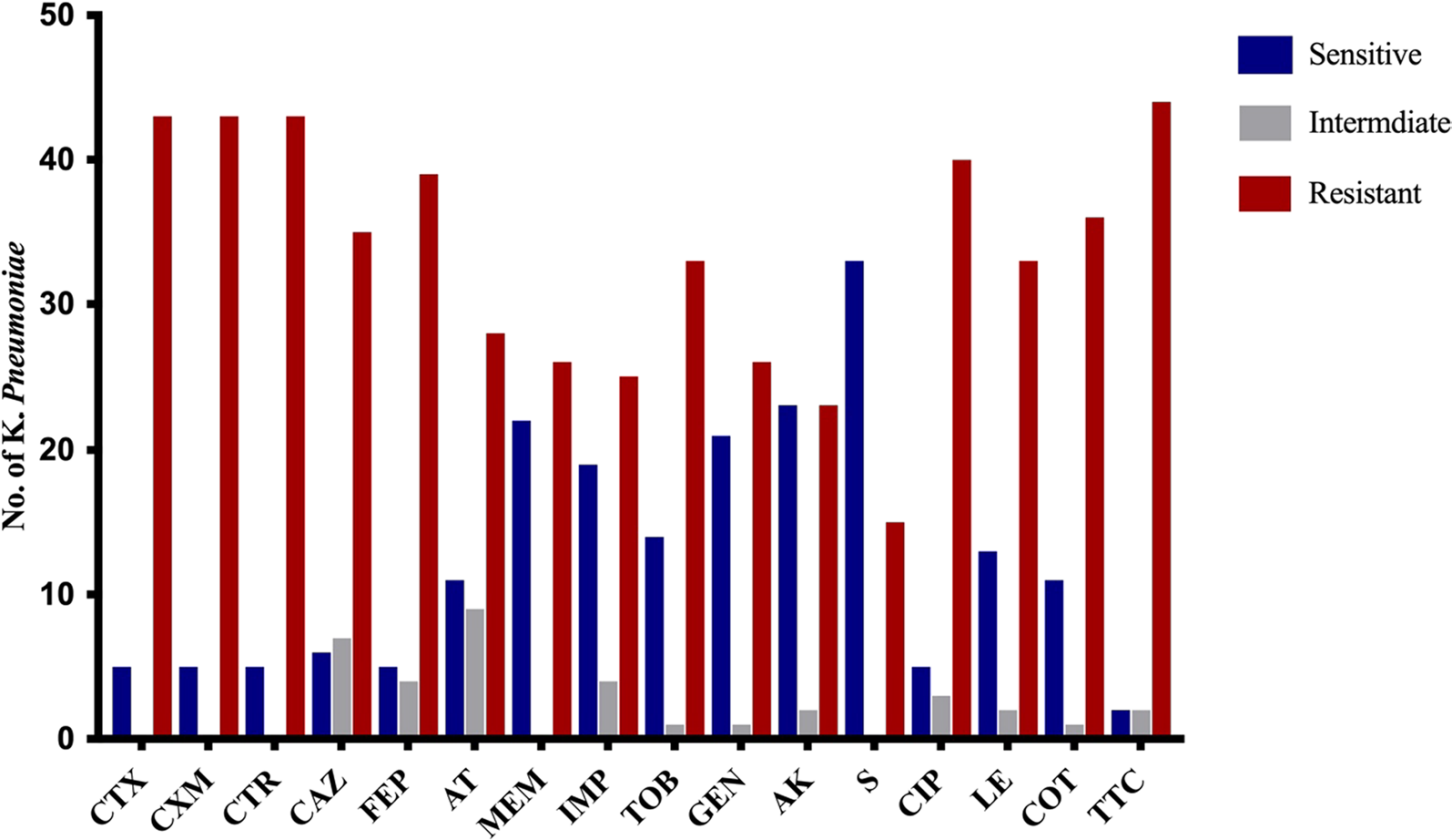
Antimicrobial susceptibility pattern of the collected *K. pneumoniae* clinical isolates*.* CTX: Cefotaxime, CXM: Cefuroxime, CTR: Ceftriaxone, CAZ: Ceftazidime, FEP: Cefepime, AT: Aztreonam, MEM: Meropenem, MP: Imipenem, TOB: Tobramycin, GEN: Gentamicin, AK: Amikacin, S: Streptomycin, CIP: Ciprofloxacin, LE: Levofloxacin, COT: Cotrimoxazole, TTC: Ticarcillin/clavulanic acid

The antimicrobial susceptibility of the collected *K. pneumoniae* isolates was assessed. Results in (Fig. 1) indicated that the highest level of resistance (91.6%) was found against ticarcillin/clavulanic acid, followed by cefuroxime, cefotaxime, and ceftriaxone (89.6%); ciprofloxacin (83.3%); cefepime (81.3%); cotrimoxazole (75%); ceftazidime (72.9%); levofloxacin and tobramycin (68.8%); aztreonam (58.3%); meropenem and gentamicin (54.2%); imipenem (52.1%); amikacin (47.9%); and streptomycin (31.3%). Based on these results, 91.7% (n = 44) of the tested isolates are multiple drug resistant. These isolates were selected for further investigations. The detailed antibiogram profile is provided in supplementary data (Table S1).

### Determination of minimum inhibitory concentrations

The MICs of gentamicin, levofloxacin, and meropenem against three of the MDR *K. pneumoniae* isolates (K22, K35, and K37) were determined (Table 1).

**Table 1:** Minimum inhibitory concentration of gentamicin, levofloxacin and meropenem against three MDR *K. pneumoniae* isolates (K22, K35, and K37)

Isolates	MIC (μg/mL)		
	Gentamicin	Levofloxacin	Meropenem
K22	250	125	125
K35	250	125	62.5
K37	250	125	62.5

### Isolation of bacteriophage

**Fig. 2:**
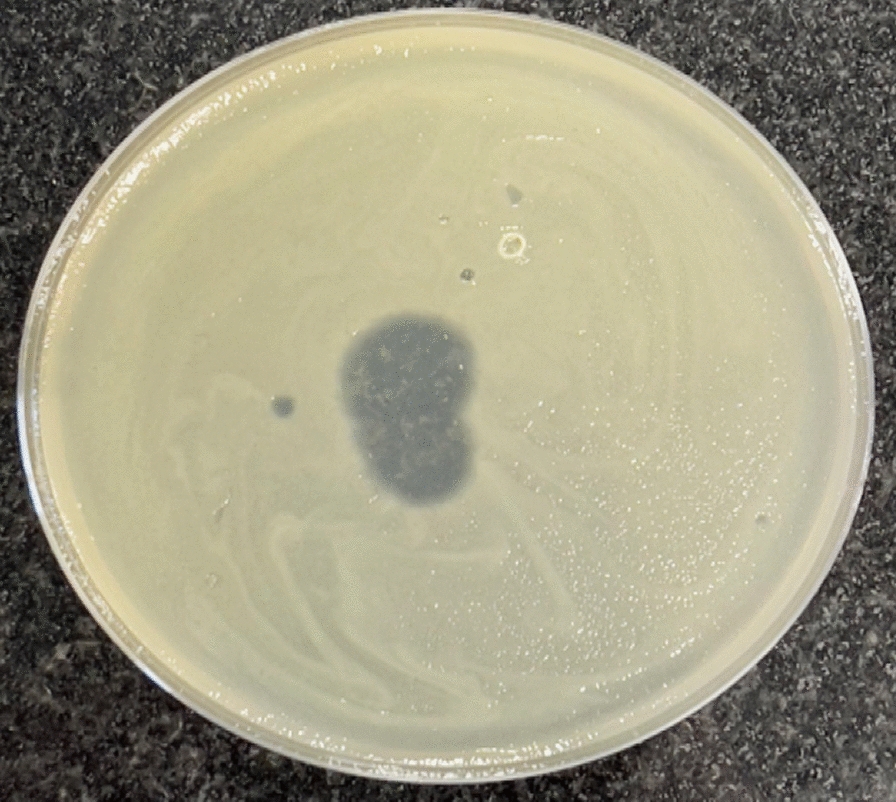
Spot test of the isolated phage showing clear spot

The obtained lysates were screened for lytic activity against MDR *K. pneumoniae* by the spot test. The screening resulted in one positive spot test and was coded as Kp25 (Fig. 2).

### Characterization of the isolated bacteriophages

#### Plaque morphology

**Fig. 3:**
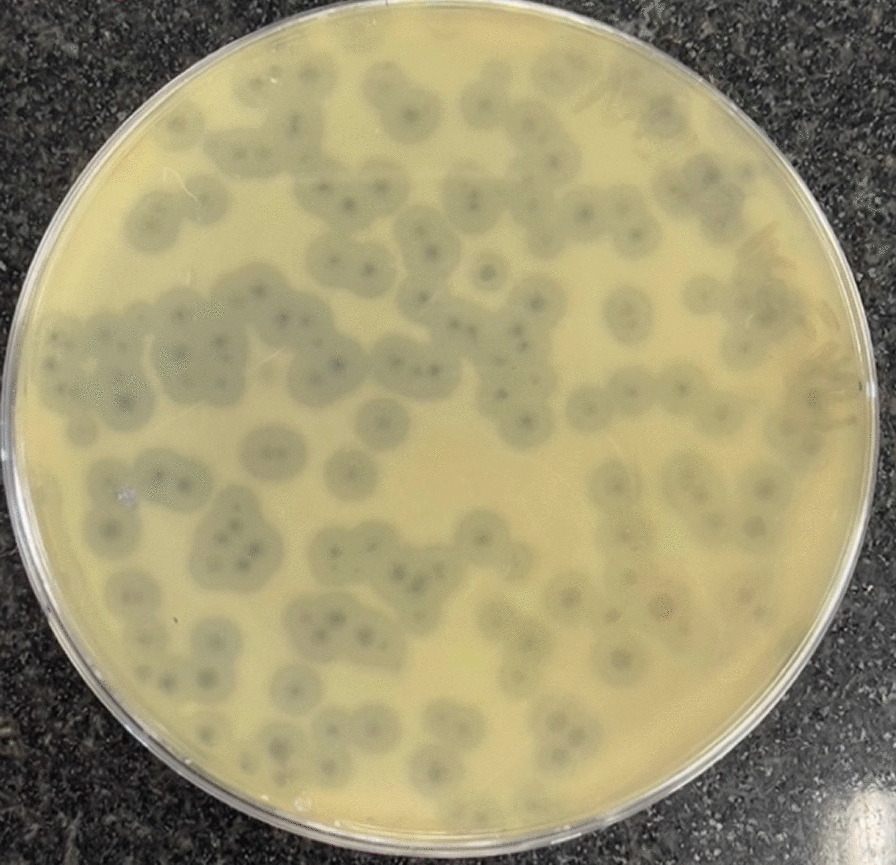
Picture of the plaques of the phage Kp25: 2 mm diameter plaques with a halo zone

The formed plaques were clear and regularly circular. The phage-produced plaque diameter was 2 mm with a halo zone (Fig. 3).

#### Determination of the host range of infection

Only 17 of the bacterial isolates provided a positive lytic effect when the phage Kp25 was observed on the surface of plates that had been previously inoculated with MDR isolates (n = 44). This information is presented in (Table 2).

**Table 2:** Host range of phage Kp25 determined on 44 MDR *K. pneumoniae* clinical isolates

Isolate	Phage	Isolate	Phage	Isolate	Phage
K1	+	K19	+	K34	−
K2	+	K20	−	K35	+
K3	−	K21	−	K36	+
K4	−	K22	+	K37	+
K5	+	K23	+	K38	−
K7	−	K24	+	K39	−
K8	−	K25	−	K40	−
K9	−	K26	+	K42	+
K10	−	K27	−	K43	+
K12	−	K28	−	K44	−
K13	−	K29	−	K45	+
K14	−	K30	+	K46	−
K15	−	K31	+	K47	−
K16	−	K32	+	K48	−
K18	−	K33	−		

Clear zone (+) and no zone (−)

#### Thermal stability

A spot test was performed to assess the thermal stability of phage Kp25 at different temperatures, and the phage retained its thermal stability when incubated at 40°C, 50°C, 60°C, 70°C, and 90°C for one hour. However, the phage was inactivated when incubated at 100°C (Table 3).

**Table 3:** Determination of thermal stability of the phage Kp25 at different temperatures

Temperature (°C)	Spot test result
40	+
50	+
60	+
70	+
90	+
100	−

(+) = lysis detected; (–) = no lysis observed)

#### pH stability

The phage Kp25 retained its activity over a wide range of pH conditions, from 3 to 11, indicating functional stability under varying environmental conditions. However, the phage was inactivated when incubated at 100°C and when incubated at stronger acidic (pH 1 and 2) and alkaline (pH 12) conditions (Table 4).

**Table 4:** Determination of pH stability of the phage Kp25 at different pH values

pH value	Spot test result
1	−
2	−
3	+
4	+
5	+
6	+
7	+
8	+
9	+
10	+
11	+
12	−

(+) indicates bacterial lysis (clear zone); (–) = no lysis observed

#### Phages longevity

The results of the longevity test for the phage Kp25 against the *K. pneumoniae* isolates demonstrate that the phage Kp25 can endure for 120 days at 4 °C and − 20 °C (Table 5).

**Table 5:** Determination of the stability of the phage Kp25 at different temperatures for 120 days by spot test

Phage code	Storage days	Storage temp (°C)	
		4	− 20
Kp25	5	+	+
	15	+	+
	30	+	+
	45	+	+
	60	+	+
	75	+	+
	90	+	+
	120	+	+

(+) indicates bacterial lysis (clear zone)

### Phage examination using transmission electron microscopy

**Fig. 4:**
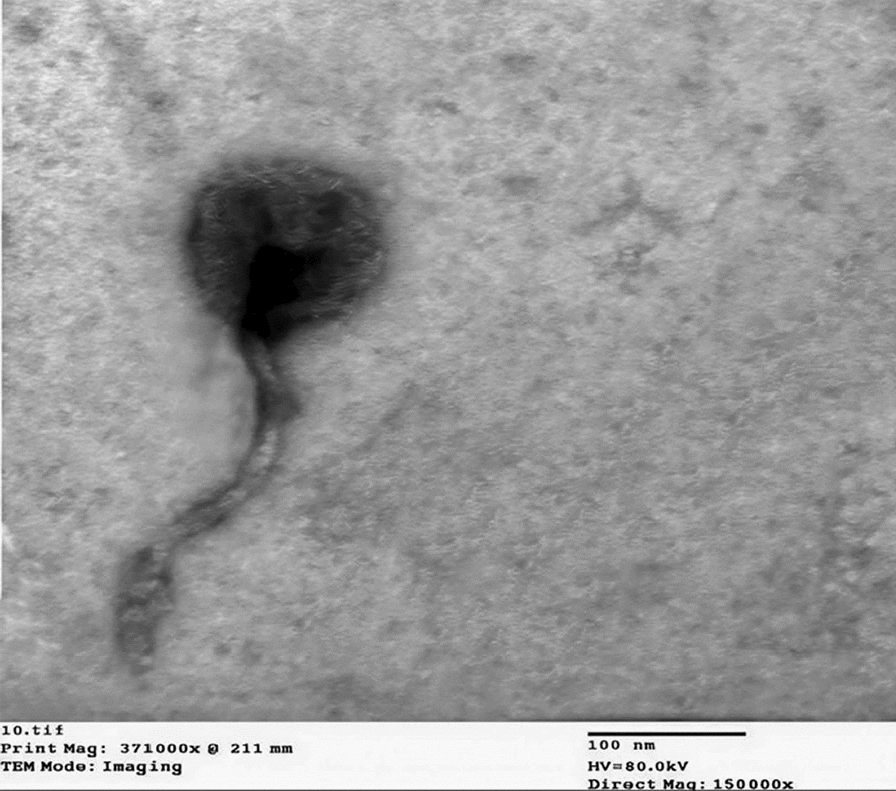
Microscopical examination of phage Kp25 by TEM. Dimension of the phage: Head 132*140 (nm) and Tail length*width 270*12 (nm)

According to data obtained from the TEM and International Committee on taxonomy of viruses, the phage contains an icosahedral head (132 × 140 nm) attached to a long non-contractile tail (270 × 12 nm), which is characteristic of the members of the order *Caudoviricetes* belonging to *Drexlerviridae* family (Fig. 4).

### Molecular analysis of the phage

**Fig. 5:**
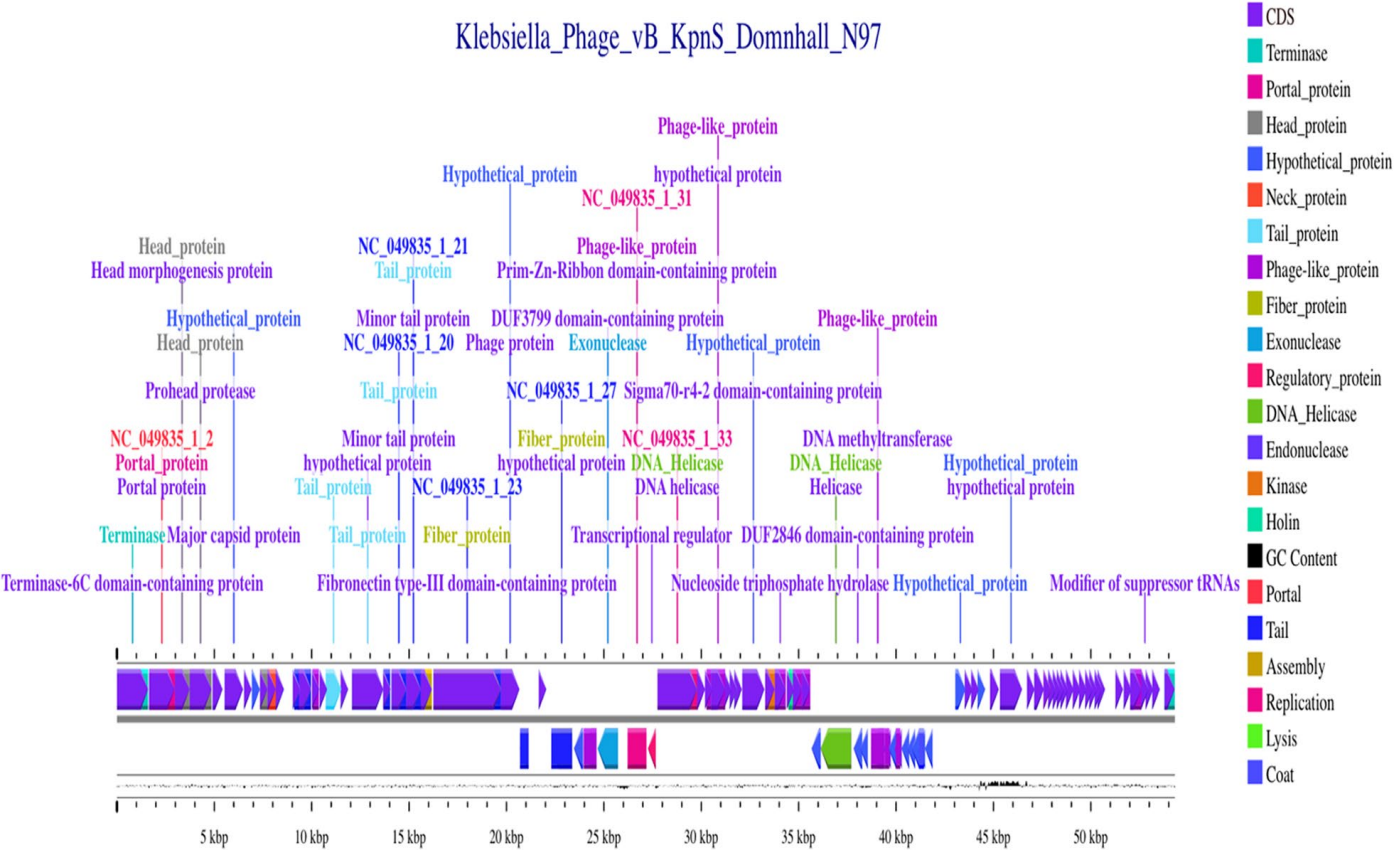
Linear genome map of vB_KpnS_domnhall_N97

The genomic sequence of the phage, which consists of a linear double-stranded DNA genome with 81 open reading frames (ORFs), has been assembled, annotated, and submitted to the NCBI GenBank database (Accession No. PV660605) https://www.ncbi.nlm.nih.gov/search/all/?term=PV660605. The genome has a GC content of 52.96% and is 54,314 base pairs long. No lysogeny-associated genes, virulence factors, or mobile genetic elements were identified in the Kp25 genome, as determined by genome annotation and thorough screening with the Proksee tool. The BLASTn alignment analysis showed the taxonomic classification of the phage, which is *Viruses; Duplodnaviria; Heunggongvirae; Uroviricota; Caudoviricetes; Drexlerviridae; Webervirus; Webervirus domnhall*. The linear genome map illustrates the functioning genes (Fig. 5).

### Determination of the optimal multiplicity of infection

**Fig. 6:**
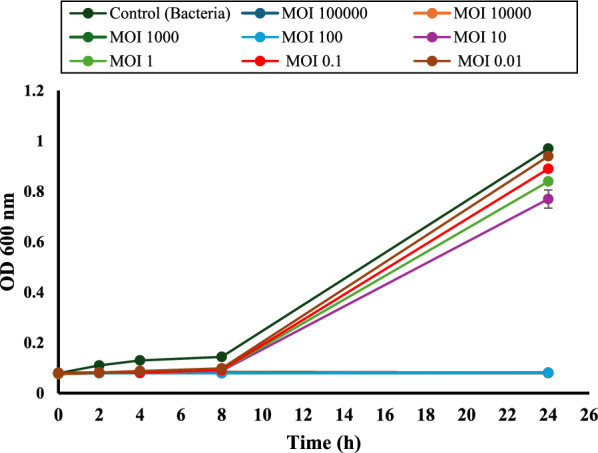
Determination of optimal multiplicity of infection at different time intervals. The experiment was performed in three biological replicates, each in triplicate. Results are presented as mean ± SD. The results of MOI 100, 1000, 10,000, and 100,000 are overlapped and appear as one line

The multiplicity of infection (MOI) was evaluated within the range of 0.01 to 100,000 in order to ascertain the optimal quantities of phage particles that were necessary to eliminate or reduce the host cell when the concentration of bacteria was 5 × 10^5^ CFU/mL. The turbidity was at its lowest point when the phage-to-bacteria ratio was 100, and for this reason, this ratio was deemed to be the best MOI. On the basis of this ideal MOI, the following studies were carried out, as shown in (Fig. 6).

### Time killing curve

**Fig. 7:**
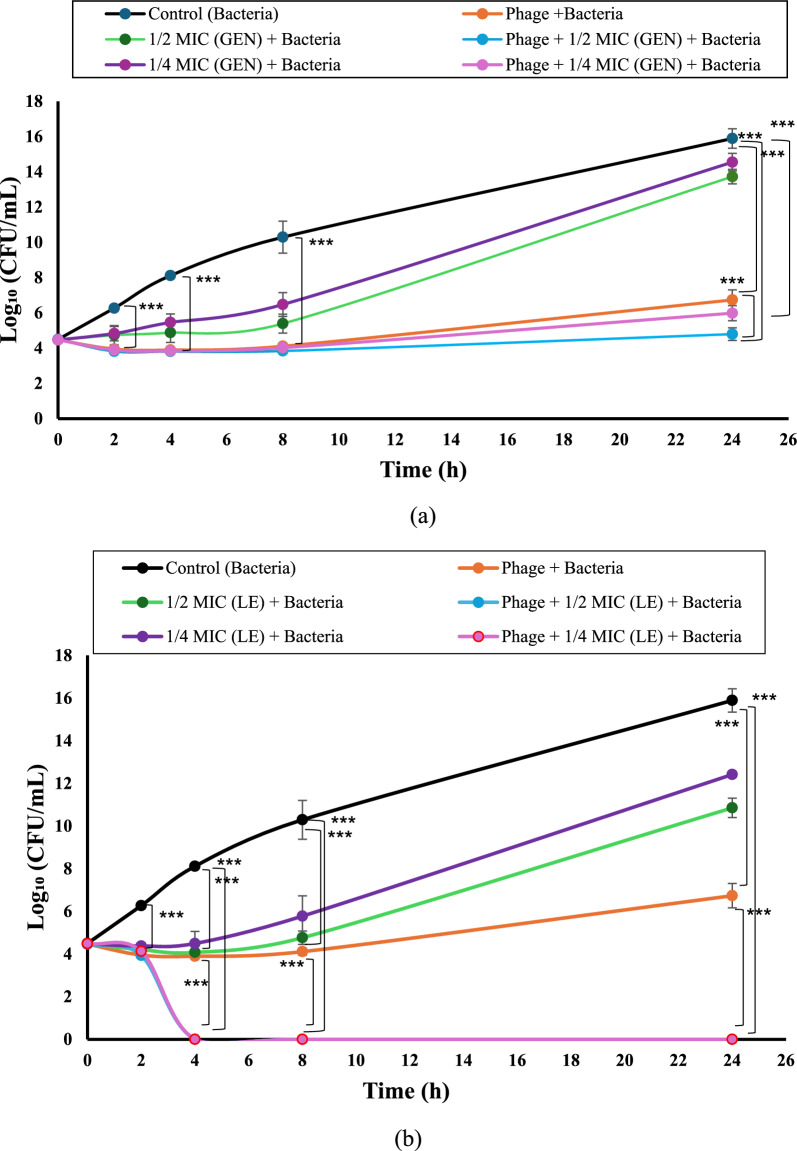

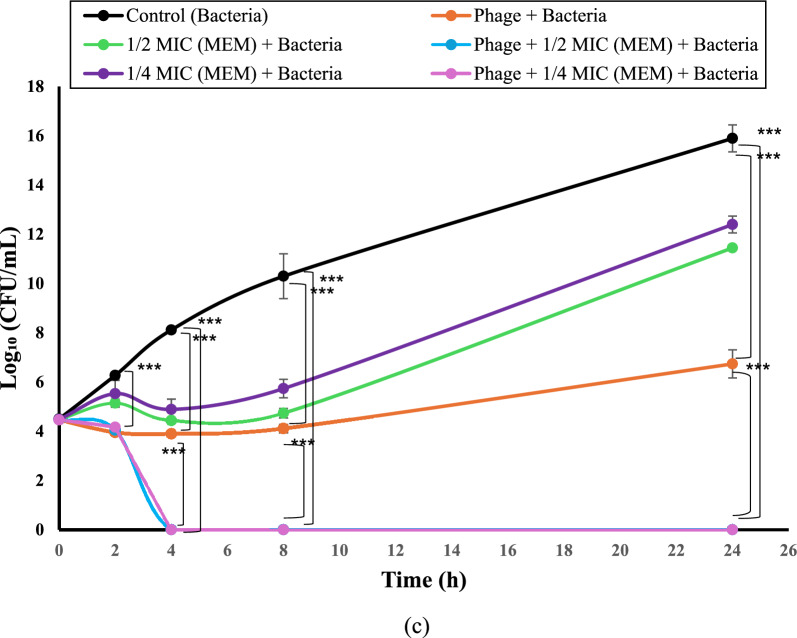
Time killing curve experiments of phage Kp25 alone and in combination with sub-MIC of antibiotics. The effect of phage alone and in combination with ½ and ¼ MIC of (**a**) Gentamicin, (**b**) Levofloxacin, and (**c**) Meropenem against three MDR *K. pneumoniae* isolates (K22, K35, and K37). The samples were taken at time intervals (0, 2, 4, 8, and 24 h incubation periods). The results of phage combination with levofloxacin and meropenem after 4 h incubation overlap and appear as one line. The experiment was performed using three independent clinical isolates, with three technical replicates per isolate. The data is presented in the form of the mean ± standard deviation, and the asterisks (***) indicate statistically significant differences (*P* < 0.001). Abbreviations: Gentamicin (GEN), Levofloxacin (LE), Meropenem (MEM), Colony Forming Unit (CFU)

Three MDR *K. pneumoniae* isolates (K22, K35, and K37) were used in this experiment to evaluate the antibacterial activity of phage alone and in combination with sub-MIC of gentamicin, levofloxacin, and meropenem. The obtained results (Fig. 7a, b, and c) showed no significant differences (*p* = 0.487) in log bacterial count between any of the groups at starting time, while at 2, 4, 8, and 24 h of incubation a significant reduction (*p* < 0.001) in log bacterial count in all treated groups (phage with or without antimicrobial agents) as compared to that of the control group was determined.

The treated groups with phage and ½ or ¼ MIC of antibiotics (gentamicin, levofloxacin, and meropenem) showed gradual reduction in log bacterial count along the first 8 h of incubation, reaching the ranges of 4–4.1, 4.7–5.4, and 5.7–6.5, respectively. Along the incubation period between 8 and 24 h, the same groups showed an increase in the bacteria count as follows: 4.1–6.7, 7.4–13.7, and 5.7–14.5, respectively (Fig. 7a, b, and c).

As compared to the phage-treated group, the combination of phage with ¼ MIC of gentamicin showed no significant difference in log bacterial count along the 24 h incubation period, while a significant reduction in the count was observed in the phage combination with ½ MIC of gentamicin at 24 h incubation (Fig. 7a).

Interestingly, the groups of phage combined with ¼ and ½ MIC of levofloxacin and meropenem showed complete in vitro eradication of bacteria at 4 h incubation without detection of survived cells along the rest of the incubation period (Fig. 7a, b, and c).

## Discussion

The emergence of antimicrobial resistance (AMR) has reached alarming levels, particularly in countries with low to middle incomes, where antibiotic overprescription and lenient pharmaceutical regulations have become widespread (Choucair et al. 2021). This growing crisis has created an urgent need for novel antimicrobial agents effective against multidrug-resistant (MDR) pathogens. Among these, *Klebsiella pneumoniae* stands out as one of the most prevalent and clinically significant MDR organisms. Its resistance to broad-spectrum antibiotics and involvement in both hospital-acquired and community infections present major challenges to current treatment approaches and public health systems globally (Balcão et al. 2022; Qadri et al. 2021).

In this context, bacteriophage therapy has come to light as an appropriate alternative or adjunct to conventional antibiotics for treating MDR *Klebsiella pneumoniae* infections, owing to its specificity and effectiveness against resistant strains. Our study investigated this approach by isolating and characterizing phage active against clinical MDR *K. pneumoniae* isolates. Of the 48 *Klebsiella pneumoniae* isolates collected, 44 (91.6%) showed multidrug resistance, with particularly high resistance to ticarcillin/clavulanic acid (91.6%). Minimum inhibitory concentration (MIC) testing revealed concerning resistance levels, with values of 250 μg/mL for gentamicin, 125 μg/mL for levofloxacin, and 62.5–125 μg/mL for meropenem across different isolates. Consequently, the sub-MIC of these antibiotics was used to perform time-kill assays, which evaluate the activity of the phage-antibiotic combination on MDR *K. pneumoniae* compared to the activity of the antibiotic and phage only.

The isolated phage Kp25 demonstrated lytic potential, forming clear circular plaques (2 mm diameter) with distinct halo zones, suggesting the production of depolymerase enzymes that degrade bacterial extracellular structures and enhance phage infectivity (Su et al. 2025). This phage exhibited activity against 17 out of 44 MDR *Klebsiella pneumoniae* isolates, corresponding to a host range of approximately 38.6%**.** This coverage is superior to that reported for many single lytic phages in recent studies. Such a relatively wide host range, accomplished without engineering or formulation, demonstrates the phage’s in vitro potential against diverse *Klebsiella* isolates, indicating a promising candidate for further therapeutic investigation.

For instance, a study recently described by Abdel-Razek et al. (2025) exhibited a notably narrower host range, lysing only 7 out of 40 tested *K. pneumoniae* isolates (17.5%), while another phage KpP-G7, was characterized by demonstrated lytic potential against 28% of clinical isolates (Rastegar et al. 2024). What distinguishes our phage is that despite being a single virion**,** it achieved broader coverage (17 isolates) than many others without genetic modification or phage cocktailing. This is likely attributed to the presence of a capsule depolymerase enzyme, as indicated by the halo-forming plaques, which facilitates penetration through diverse capsular types, a major barrier in *Klebsiella* phage therapy (Concha-Eloko et al. 2023).

Stability testing revealed that phage Kp25 retained its lytic activity even at high levels of temperatures up to 90 °C, which suggests that the phage possesses heat-stable structural protein or protective conformational features that could sustain its morphology, resilience, and efficacy. Consequently, the isolated phage Kp25 may withstand processes utilized in the production of formulations like aerosols and have a favorable shelf life (Pradeep et al. 2022). The thermal durability exceeds what is typically reported for phages, many of which lose viability at 70–80 °C (Li et al. 2020). As for longevity, all phages remained viable for 120 days when stored at 4 °C and − 20 °C (Fayez et al. 2023). Moreover, phage Kp25 resistance at severe pH values that inhibit phage denaturation through irreversible precipitation and coagulation. This aligns closely with findings by Li et al. (2021) who reported similar stability in phage vB_Kox_ZX8. These findings provide additional confidence regarding the phage's potential for therapeutic development. This broad pH tolerance supports the feasibility of oral or inhalation formulations and underscores the isolate’s suitability for environments with variable pH, such as the gastrointestinal tract or infected tissues (Malik et al. 2017). The combination of these characteristics suggests that phage Kp25 may serve as a basis for further studies and could inform the development of pharmaceutical formulations or industrial applications, pending additional validation**.**

The intrinsic durability of the substance suggests that it offers practical advantages in formulation, storage, and distribution, particularly in environments with limited resources. Furthermore, the adaptability of Kp25 to difficult clinical settings is demonstrated by the fact that it is able to maintain its viability in the face of significant physical and chemical stressors. It is possible that this resistance could be advantageous when it comes to treating illnesses that are located in areas that are less accessible or unstable, where other phages could fail.

A methodological limitation of this work is that the thermal and pH stability assays were conducted qualitatively without PFU quantification. Future studies should include quantitative plaque assays to provide more reliable stability data under various conditions.

Microscopical analysis by TEM confirmed that the isolated phage Kp25 belongs to the *Drexlerviridae* family. Genomic characterization revealed a 54,314 bp genome with 52.96% GC content, containing 81 open reading frames (ORFs), which has been deposited in the NCBI GenBank database (Accession No. PV660605). The phage vB_KpnS-FZ10, described by Zurabov and Zhilenkov (2021), belongs to the *Drexlerviridae* family, genus *Webervirus*. It possesses a 50,381 bp double-stranded DNA genome with a GC content of 50.66% and encodes 42 predicted ORFs**.** The genome is ~ 4 kb larger than *vB KpnS FZ10* yet remains within the 50 ± 5 kb envelope that typifies *Weberviruses*, while encoding nearly double the ORF complement of FZ10. This higher coding capacity may explain the pronounced capsule depolymerase halo and the broader host range we observed (Zurabov and Zhilenkov 2021). *Viruses; Duplodnaviria; Heunggongvirae; Uroviricota; Caudoviricetes; Drexlerviridae; Webervirus; Webervirus domnhall* were the additional taxonomical classifications that were assigned to the phage through the use of the BLASTn alignment technique. The characterization of novel phages is enhanced by the use of whole genome analysis, which makes it possible to effectively overcome the shortcomings of in vitro research. Based on the findings of the genomic research, a cluster of two genes that are likely to encode holin and lysozyme was discovered. Holins are responsible for the formation of apertures in the inner membrane of the cytoplasmic membrane of bacteria. These apertures promote the movement of lysozymes into the periplasmic region, which in turn enables the lysozymes to successfully destroy the peptidoglycan barrier that is present in many bacteria (Philipson et al. 2018). The predicted clustering of genes coding for holin and lysozyme suggests the probable mechanism through which Kp25 lyses infected bacterial cells to release freshly generated offspring at the conclusion of the lytic cycle.

The growing global threat of multidrug-resistant *Klebsiella pneumoniae* demands innovative therapeutic approaches beyond conventional antibiotics. This study investigation of phage-antibiotic synergy (PAS) demonstrated auspicious results. Time-kill assays revealed that combining our phage with sub-MIC concentrations (½ and ¼ MIC) of levofloxacin or meropenem achieved complete in vitro eradication of bacterial cells after 4 h incubation under the specific laboratory conditions tested (*p* < 0.001), aligning with the observations reported by Zhao et al. 2024), who noted enhanced bactericidal effects when using phage–antibiotic combinations under similar in vitro conditions. However, these results contrast with Xu et al. (2022), who reported no synergy between single phages and meropenem, highlighting the importance of phage selection in combination therapies.

Interestingly, the gentamicin-phage combination exhibited bacteriostatic rather than bactericidal effects (*p* < 0.001), with bacterial regrowth observed after 8 h incubation, which is matched with results reported by Sundaramoorthy et al. (2021). The bacterial regrowth observed may result from several interconnected mechanisms. The most common explanation is the emergence of phage-resistant mutants through receptor modification, phase variation, or insertion sequence activity that prevents further phage adsorption. In addition, a fraction of the bacterial population may enter a transient tolerant or persister state, enabling survival during phage or antibiotic exposure and subsequent regrowth once the selective pressure decreases (Bleriot et al. 2024). Biofilm-associated protection and population heterogeneity could also contribute, as spatial structure and nutrient gradients can limit phage access to deeper layers, allowing regrowth of surviving cells. Furthermore, suboptimal multiplicity of infection (MOI) over time, resulting from phage inactivation or adsorption imbalance, may reduce effective killing efficiency. Future studies should perform serial-passage resistance monitoring, whole-genome sequencing of survivors, and dynamic infection modeling to elucidate these processes and optimize phage–antibiotic combination protocols (Oechslin 2018). This differential activity highlights the antibiotic-specific nature of phage synergies, which may be related to distinct. Such differences underline the sophistication of phage-antibiotic dynamics and the significance of studying antibiotic-phage-bacteria interactions in greater depth. This diversity in response further underlines the necessity for tailored combination regimens depending on both the phage and the antibiotic profile.

The study findings support the growing consensus that, under in vitro conditions, combination therapies often surpass monotherapy in effectiveness against MDR pathogens. This work provides evidence for a combination of phage and antibiotic as an innovative approach to overcome MDR *Klebsiella pneumoniae-*related diseases, potentially enabling reduced antibiotic doses while increasing treatment efficacy. The observed variations in synergy between different antibiotics emphasize the need for careful selection of combination partners, suggesting that optimal therapeutic regimens may require personalized approaches based on both bacterial and phage characteristics.

While bacteriophages have demonstrated antimicrobial activity against bacteria, many mechanistic aspects remain to be fully elucidated. Further research should focus on understanding the molecular basis of phage-antibiotic interactions, the development of long-term resistance, and the clinical translation of these promising laboratory findings. Thus, a clear road map is recommended to support the development of phage-antibiotic combination therapy. This includes the implementation of in vivo studies to confirm the promising efficacy of the combined approach. Further mechanistic investigations are needed to elucidate the mechanism of action of the phage-antibiotic combination therapy against MDR bacteria. Additionally, formulation development should focus on designing an appropriate delivery system such as topical preparations. Pilot clinical studies involving human participants to evaluate toxicity and ensure safety. Addressing these stages will contribute to the optimization of a novel, effective phage-based therapeutic capable of combating and reducing the threat of resistant bacterial infections.

## Supplementary Information

Supplementary Material 1.

## Data Availability

The published manuscript contains full descriptions of all of the data that was gathered and analyzed throughout the present investigation.

## Abbreviations

AK: Amikacin
AMR: Antimicrobial resistance
AT: Aztreonam
BLAST: Basic local alignment search tool
CAZ: Ceftazidime
CFU: Colony forming unit
CIP: Ciprofloxacin
CLSI: Clinical and laboratory standards institute
COT: Cotrimoxazole
CTR: Ceftriaxone
CTX: Cefotaxime
CXM: Cefuroxime
FEP: Cefepime
GEN: Gentamicin
ICTV: International committee taxonomy of viruses
LE: Levofloxacin
MDR: Multidrug resistant
MEM: Meropenem
MHA: Mueller Hinton Agar
MIC: Minimum inhibitory concentration
MOI: Multiplicity of infection
MP: Imipenem
OD: Optical density
ORFs: Open reading frames
PFU: Plaque forming unit
Phage: Bacteriophage
S: Streptomycin
TEM: Transmission electron microscopy
TOB: Tobramycin
TSA: Tryptic soy agar
TTC: Ticarcillin/clavulanic acid
WDCM: World data centre for microorganisms
