# Supplementary material for: Phage-antibiotic combination: a possible approach to combatting multidrug-resistant Klebsiella pneumoniae
Source: AMB Express. 2026 Mar 25;16:38. doi: 10.1186/s13568-025-02005-1 (PMC13065991; doi:10.1186/s13568-025-02005-1)
Supplement: Supplementary file 1 — Supplementary Material 1. [file 13568_2025_2005_MOESM1_ESM.docx]

Table S1. The susceptibility of the collected *Klebsiella* *pneumoniae* clinical isolates to different antimicrobial agents

| **Isolate Code** | **Antimicrobial agents**  **(Inhibition zone in mm)** | | | | | | | | | | | | | | | | |
| --- | --- | --- | --- | --- | --- | --- | --- | --- | --- | --- | --- | --- | --- | --- | --- | --- | --- |
|  | **CTX** | **CXM** | | **CTR** | **CAZ** | **FEP** | **AT** | **MEM** | **IMP** | **TOB** | **GEN** | **AK** | **S** | **CIP** | **LE** | **COT** | **TTC** |
| K1 | 11  R | | zero  R | 7  R | 15  R | 14  R | 19  I | 30  S | 27  S | 8  R | 21  S | 20  S | 7  R | Zero  R | Zero  R | 9  R | Zero  R |
| K2 | 15  R | zero  R | | 14  R | 18  I | 12  R | 18  I | 30  S | 28  S | 8  R | 18  S | 20  S | 8  R | 9  R | 18  I | 19  S | Zero  R |
| K3 | zero  R | zero  R | | zero  R | Zero  R | Zero  R | Zero  R | 8  R | 12  R | 11  R | 21  S | 17  S | 18  S | 8  R | 10  R | 21  S | Zero  R |
| K4 | 7  R | zero  R | | 6  R | Zero  R | 15  R | 21  S | 16  R | 16  R | 12  R | 18  S | 19  S | 19  S | 20  R | 25  S | Zero  R | Zero  R |
| K5 | 34  S | 25  S | | 30  S | 22  S | 28  S | 31  S | 30  S | 27  S | 18  S | 13  I | 21  S | 10  R | 7  R | 15  R | 18  S | Zero  R |
| K6 | 33  S | 26  S | | 30  S | 29  S | 30  S | 35  S | 30  S | 26  S | 17  S | 20  S | 21  S | 16  S | 30  S | 30  S | 31  S | 17  I |
| K7 | zero  R | Zero  R | | zero  R | Zero  R | Zero  R | 9  R | 6  R | 14  R | Zero  R | Zero  R | Zero  R | 20  R | Zero  R | Zero  R | Zero  R | Zero  R |
| K8 | 11  R | Zero  R | | 10  R | 18  I | 17  R | 21  S | 30  S | 26  S | 9  R | 20  S | 21  S | 16  S | 11  R | 19  I | 11  R | Zero  R |
| K9 | 10  R | Zero  R | | 11  R | 26  S | 16  R | 20  I | 30  S | 29  S | 15  S | 11  R | 20  S | 6  R | 29  S | 28  S | 9  R | 9  R |
| K10 | zero  R | Zero  R | | Zero  R | 7  R | Zero  R | 7  R | 23  S | 26  S | Zero  R | Zero  R | Zero  R | 19  S | Zero  R | 8  R | 9  R | Zero  R |
| K11 | 34  S | 29  S | | 31  S | 28  S | 32  S | 35  S | 33  S | 29  S | 20  S | 21  S | 22  S | 20  S | 33  S | 30  S | 34  S | 20  S |
| K12 | zero  R | Zero  R | | zero  R | Zero  R | Zero  R | 16  R | 7  R | 14  R | Zero  R | Zero  R | Zero  R | 18  S | 11  R | 9  R | Zero  R | Zero  R |
| K13 | zero  R | zero  R | | zero  R | Zero  R | Zero  R | 15  R | 10  R | 16  R | Zero  R | Zero  R | Zero  R | 20  S | 10  R | 9  R | Zero  R | Zero  R |
| K14 | zero  R | zero  R | | zero  R | Zero  R | Zero  R | 16  R | 7  R | 14  R | Zero  R | Zero  R | Zero  R | 20  S | 11  R | 11  R | Zero  R | Zero  R |
| K15 | zero  R | zero  R | | zero  R | Zero  R | Zero  R | 15  R | 7  R | 15  R | Zero  R | Zero  R | Zero  R | 18  S | 7  R | 10  R | Zero  R | Zero  R |
| K16 | zero  R | zero  R | | zero  R | Zero  R | Zero  R | 12  R | 7  R | 12  R | Zero  R | Zero  R | Zero  R | 17  S | Zero  R | 13  R | Zero  R | Zero  R |
| K17 | 34  S | 29  S | | 33  S | 30  S | 33  S | 35  S | 33  S | 30  S | 18  S | 23  S | 22  S | 20  S | 32  S | 31  S | 31  S | 20  S |
| K18 | 15  R | 9  R | | 12  R | 20  I | 18  R | 24  S | 32  S | 30  S | 10  R | 21  S | 20  S | 19  S | 14  R | 20  R | 14  I | 10  R |
| K19 | 11  R | zero  R | | 7  R | 17  R | 14  R | 19  I | 23  S | 20  I | 12  R | 22  S | 21  S | 17  S | Zero  R | 9  R | 10  R | Zero  R |
| K20 | zero  R | zero  R | | zero  R | Zero  R | 7  R | 16  R | 7  R | 12  R | Zero  R | Zero  R | Zero  R | 20  S | 9  R | 11  R | Zero  R | Zero  R |
| K21 | 13  R | zero  R | | 12  R | 16  R | 17  R | 20  I | 32  S | 31  S | 9  R | 12  R | 20  S | 10  R | 9  R | 15  R | Zero  R | Zero  R |
| K22 | zero  R | zero  R | | zero  R | Zero  R | 7  R | 12  R | 8  R | 14  R | Zero  R | Zero  R | Zero  R | 20  S | Zero  R | 8  R | Zero  R | Zero  R |
| K23 | Zero  R | zero  R | | zero  R | Zero  R | Zero  R | 12  R | 7  R | 12  R | Zero  R | Zero  R | Zero  R | 16  S | Zero  R | 8  R | Zero  R | Zero  R |
| K24 | Zero  R | zero  R | | zero  R | Zero  R | Zero  R | 17  R | Zero  R | 11  R | Zero  R | Zero  R | Zero  R | 16  S | Zero  R | Zero  R | Zero  R | Zero  R |
| K25 | Zero  R | zero  R | | zero  R | Zero  R | 8  R | 16  R | 9  R | 13  R | Zero  R | Zero  R | Zero  R | 19  S | Zero  R | 6  R | Zero  R | Zero  R |
| K26 | Zero  R | zero  R | | zero  R | Zero  R | 7  R | 16  R | 9  R | 14  R | Zero  R | Zero  R | Zero  R | 20  S | Zero  R | Zero  R | Zero  R | Zero  R |
| K27 | Zero  R | zero  R | | zero  R | 19  I | 7  R | 18  I | 9  R | 15  R | Zero  R | Zero  R | Zero  R | 21  S | Zero  R | 10  R | Zero  R | Zero  R |
| K28 | 9  R | zero  R | | 11  R | 15  R | 17  R | 16  R | 23  S | 21  I | 23  S | 24  S | 15  I | 10  R | 21  R | 21  S | 21  S | Zero  R |
| K29 | 12  R | zero  R | | 14  R | 19  I | 20  I | 21  S | 35  S | 30  S | 21  S | 20  S | 19  S | 7  R | 25  I | 25  S | 26  S | 7  R |
| K30 | Zero  R | zero  R | | zero  R | Zero  R | 7  R | 12  R | 11  R | 15  R | 9  R | Zero  R | Zero  R | 19  S | 8  R | 11  R | Zero  R | Zero  R |
| K31 | 14  R | 9  R | | 16 R | 16  R | 18  R | 14  R | 25  S | 30  S | 14  I | 15  S | 24  S | 16  S | Zero  R | 15  R | Zero  R | 14  R |
| K32 | Zero  R | zero  R | | zero  R | Zero  R | 6  R | 14  R | 8  R | 14  R | Zero  R | Zero  R | Zero  R | 20  S | Zero  R | Zero  R | Zero  R | Zero  R |
| K33 | Zero  R | zero  R | | zero  R | Zero  R | Zero  R | 12  R | 8  R | 13  R | 11  R | 24  S | 14  R | 20  S | Zero  R | Zero  R | 11  R | Zero  R |
| K34 | 17  R | zero  R | | 12  R | 20  I | 20  R | 22  S | 35  S | 30  S | 11  R | 11  R | 22  S | 11  R | Zero  R | 16  R | 7  R | 11  R |
| K35 | Zero  R | zero  R | | zero  R | Zero  R | Zero  R | 13  R | 9  R | 14  R | 35  R | Zero  R | Zero  R | 20  S | Zero  R | Zero  R | Zero  R | Zero  R |
| K36 | Zero  R | zero  R | | zero  R | Zero  R | Zero  R | 17  R | Zero  R | 11  R | 8  R | 21  S | 12  R | 17  S | Zero  R | 7  R | Zero  R | Zero  R |
| K37 | Zero  R | zero  R | | zero  R | Zero  R | Zero  R | 17  R | 7  R | 11  R | Zero  R | Zero  R | Zero  R | 18  S | Zero  R | 7  R | 24  S | Zero  R |
| K38 | Zero  R | zero  R | | zero  R | 10  R | Zero  R | 14  R | 16  R | 20  I | 19  S | 10  R | 25  S | 23  S | 15  R | 21  S | 38  S | Zero  R |
| K39 | Zero  R | zero  R | | zero  R | Zero  R | Zero  R | 7  R | 15  R | 19  R | Zero  R | Zero  R | Zero  R | 22  S | 12  R | 14  R | Zero  R | Zero  R |
| K40 | 14  R | zero  R | | 12  R | 20  I | 19  I | 23  S | 33  S | 23  S | 29  S | 25  S | 22  S | 11  R | 34  S | 32  S | 8  R | 11  R |
| K41 | 35  S | 25  S | | 33  S | 26  S | 34  S | 35  S | 35  S | 30  S | 20  S | 24  S | 22  S | 20  S | 25  I | 24  S | 20  S | 15  I |
| K42 | 10  R | zero  R | | 13  R | 16  R | 21  I | 19  I | 35  S | 29  S | 28  S | 21  S | 21  S | Zero  R | 21  R | 25  S | Zero  R | Zero  R |
| K43 | 10  R | zero  R | | 9  R | 14  R | 13  R | 19  I | 25  S | 22  I | 20  S | 21  S | 21  S | Zero  R | 22  I | 24  S | Zero  R | Zero  R |
| K44 | 13  R | zero  R | | 15  R | 14  R | 21  I | 17  R | 33  S | 30  S | 11  R | 11  R | 20  S | 11  R | 7  R | 14  R | Zero  R | Zero  R |
| K45 | 9  R | zero  R | | 11  R | 16  R | 16  R | 20  I | 31  S | 27  S | 19  S | 20  S | 20  S | 10  R | 20  R | 24  S | 7  R | Zero  R |
| K46 | Zero  R | zero  R | | zero  R | Zero  R | Zero  R | Zero  R | Zero  R | 11  R | 10  R | Zero  R | Zero  R | 10  R | Zero  R | 8  R | Zero  R | Zero  R |
| K47 | Zero  R | zero  R | | zero  R | Zero  R | Zero  R | 10  R | 8  R | 12  R | 20  S | 19  S | 15  I | 18  S | Zero  R | Zero  R | Zero  R | Zero  R |
| K48 | Zero  R | zero  R | | zero  R | 14  R | Zero  R | Zero  R | Zero  R | 9  R | 9  R | Zero  R | Zero  R | 10  R | Zero  R | 8  R | Zero  R | Zero  R |

mm= millimeter

R: Resistant, I: Intermediate, S: Sensitive, according to CLSI, 2021

Abbreviations: CTX: Cefotaxime, CXM: Cefuroxime, CTR: Ceftriaxone, CAZ: Ceftazidime, FEP: Cefepime, AT: Aztreonam, MEM: Meropenem, MP: Imipenem, TOB: Tobramycin, GEN: Gentamicin, AK: Amikacin, S: Streptomycin, CIP: Ciprofloxacin, LE: Levofloxacin, COT: Cotrimoxazole, TTC: Ticarcillin/clavulanic acid.
